# Nutrition for Brain Development

**DOI:** 10.3390/nu14071419

**Published:** 2022-03-29

**Authors:** M. Hasan Mohajeri

**Affiliations:** Department of Human Medicine, University of Zurich, Winterthurerstrasse 190, 8057 Zürich, Switzerland; mhasan.mohajeri@uzh.ch; Tel.: +41-79-938-1203

This Special Issue focuses on the fundamental role of nutrition in brain development. A steady and sufficient supply of oxygen and dietary ingredients are indispensable for proper brain functioning but genetic and environmental factors may influence brain development and function. Imbalance in any of these factors may lead to the manifestation of developmental disorders of young ages, compromised daily capabilities, or age-associated brain disorders. This editorial will focus on important topics discussed in individual reports included in this Special Issue.

Granziera et al. [1] studied the associations between habitual food consumption, body mass index (BMI), and cognitive outcomes in 54 preschool children born in 2011–2014 and living in Tuscany, Italy. These authors showed, by using the Griffiths Mental Development Scales-Extended Revised (GMDS-ER) test, that adherence to the Mediterranean diet was associated with higher cognitive scores. Importantly, a high body mass index negatively impacted cognition. All associations were independent of maternal IQ, socioeconomic status, breastfeeding, actual age at cognitive assessment, and gender. 

Nuthikattu and colleagues [2] showed, using a multi-omic approach, that a high glycemic diet (HGD) leads to differential expression of 608 genes in vivo. HGD affected gene expression of brain microvessels in memory centers by up-regulating the protein-coding and non-coding genes involved in mitochondrial function, oxidation, inflammation, and microvascular functioning. This report showed that inhibition of soluble epoxide hydrolase protects against cognitive decline by down-regulating the above-mentioned differentially expressed genes up-regulated by HGD.

The effects of spearmint extract (SME) and rosmarinic acid (the major component of SME) were examined on the amyloid fibril formation of αSyn, Aβ, and Tau proteins in vitro [3]. Utilizing thioflavin T (ThioT) binding assays and transmission electron microscopy (TEM), it was concluded that rosmarinic acid could disassemble preformed fibrils of αSyn, Aβ, and Tau. Given the fact that a successful therapy for neurodegenerative disorders has not been developed despite decades of intensive research [4], rosmarinic acid may be a promising candidate to be tested in disease models of amyloidosis and supports the notion that dietary ingredients may exhibit a realistic potential to improve brain functions in vivo [5].

Dietary restriction is known to profoundly affect fetal brain development. The report by Frapin et al. asked the question as to which are the cellular and molecular systems underlying the effects of maternal protein restriction (MPR) during fetal development [6]. Transcriptomic analysis of the fetal rat hypothalamus revealed that some genes encoding proteins of the mitochondrial respiratory chain were overexpressed and the mitochondrial metabolic activity in the fetal hypothalamus was altered. Collectively, this report suggests that MPR leads to early alterations of neuronal development and subsequent impaired hypothalamus function in vivo.

In their study, Gawliński et al. evaluated how maternal diet determines the reinstatement of cocaine-seeking behavior and the expression of melanocortin-4 receptors in female rat offspring [7]. The authors showed that a maternal high-sugar diet is an important factor that triggers cocaine-seeking behavior in female offspring and the expression of melanocortin-4 (MC-4) receptors in the nucleus accumbens. Moreover, they suggested that an altered amount of macronutrients in the maternal diet disrupts the proper expression of MC-4 receptors in brain structures involved in cocaine relapse. 

In their elaborated review, Kovacs et al. examined the potential beneficial effects of ketogenic supplements on the aging process and age-related neurodegenerative diseases. They concluded that exogenous ketogenic supplements (EKS), such as ketone salts and ketone esters, may mitigate aging processes, delay the onset of age-associated diseases and extend lifespan through ketosis. Consequently, the administration of EKS may be a potential therapeutic tool as an adjuvant therapeutics in combination with therapeutic drugs against age-related neurodegenerative diseases and increase the health span of the aging human population [8].

A collaborative effort of scientists in the United Kingdom and Italy [9] compared the test batteries, designed to monitor the effect of phenylketonuria (PKU) on cognitive performance. The parameter in the focus of this study included visual attention, visuomotor coordination, executive functions, sustained attention, verbal and visual memory, and learning. The results suggested that batteries with the same and/or matched tasks can be used to assess cognitive outcomes across countries allowing results to be compared and accrued.

Manganese (Mn) is a trace nutrient necessary for life but is toxic to the brain at high concentrations. McCabe and Zhao [10] provided an insight into the transport mechanisms of Mn through the blood–brain barrier (BBB) and the blood–CSF barrier (BCB) and its hemostasis in the brain by reviewing in vitro and in vivo models. 

The potential effects of the human milk oligosaccharides (HMO) on cognitive functions were reviewed in mice, rats, and piglets [11]. The authors concluded that the administration of fucosylated (single or combined with Lacto-N-neoTetraose and other oligosaccharides) and sialylated HMOs results in marked age-dependent improvements in spatial memory and in an accelerated learning rate in operant tasks, which already become apparent during infancy. A combination of HMOs with other oligosaccharides yielded different effects on memory performance as opposed to single HMO administration, a topic that is being intensively researched. 

Lastly, we evaluated in a systematic review [12] the available preclinical and clinical data on alterations of the gut microbiome, particularly on low taxonomic levels, and related them to the pathophysiology of major depressive (MDD) and bipolar disorder (BD). A discussion of diagnostic and treatment response parameters, their health-promoting potential, as well as novel adjunctive treatment options are also discussed. We also take on the task of systematically evaluating the role of the bacterial metabolites, beyond the short-chain fatty acids (SCFA), in brain development and different neurodegenerative diseases [13]. SCFA are extensively studied in various test systems, but the biology of other bacterial metabolites in health and disease is an overtly under-researched topic. Our data highlight the existence of altered bacterial metabolites in patients across various brain diseases and describe protective and detrimental effects of some bacterial metabolites in brain diseases such as autism spectrum disorder, affective disorders, multiple sclerosis, and Parkinson’s disease. These findings could lead to further insights into the gut–brain axis and thus into potential diagnostic, therapeutic, or preventive strategies in brain diseases.

In conclusion, diet is of fundamental importance for the development of the brain. Given that diet directly or indirectly affects brain development and function, carefully planned and masterfully conducted basic and clinical research is needed to understand brain development better and to answer the question to which extent diet-related strategies can prevent brain disorders or be therapeutically exploited. 

## Data Availability

Not applicable.
